# Development of Microemulsion for Solubility Enhancement of Clopidogrel

**Published:** 2010

**Authors:** Vandana Patel, Hirenkumar Kukadiya, Rajshree Mashru, Naazneen Surti, Surjyanarayan Mandal

**Affiliations:** *Baroda college of Pharmacy, Gujarat, India.*

**Keywords:** Clopidogrel, Microemulsion, Solubility, Zeta potential, Phase diagrams

## Abstract

Clopidogrel, an inhibitor of platelet aggregation, selectively inhibits the binding of adenosine diphosphate (ADP) to its platelet receptor and the subsequent ADP-mediated activation of the glycoprotein GPIIb/IIIa complex, thereby inhibiting platelet aggregation. Oral bioavailability of clopidogrel is very low (less than 50%), due to its poor water solubility. The aim of this investigation was to design and develop a microemulsion formulation of clopidogrel for enhancing its solubility, and hence its oral bioavailability. For this purpose, initially, solubility of clopidogrel was determined in various vehicles. Next, pseudo-ternary phase diagrams were constructed to identify the microemulsion existing zone. Solubility study was also performed for optimization of formulation. The optimized microemulsion formulation was characterized for its transparency, droplet size, zeta potential, viscosity, conductivity, % assay, and phase separation study. Particle size and zeta potential of the optimized microemulsion formulation were found to be 12.3 nm, and -6.34 mV, respectively. The viscosity and conductivity data indicated that the microemulsion was of the o/w type. Solubility of clopidogrel was successfully enhanced by 80.66 times, via capmul microemulsion, compared with distilled water (pH = 7.4). 75.53% and 71.2 % of the drug content were found to be released within 9 h in the *in-vitro *and *ex-vivo *studies, respectively. Hence, by formulating into microemulsion, the solubility of clopidogrel was found to be significantly enhanced.

## Introduction

Successful oral delivery of drugs has always remained a challenge to the drug delivery field, since approximately 40% of the new drug candidates have poor water solubility, and thus oral delivery is frequently associated with implications of low bioavailability. To overcome these bioavailability problems, various formulation strategies have been reported including the use of surfactants, cyclodextrin inclusion complexes, solid dispersions, nanoparticles and absorption enhancers. However, the most appropriate formulation and their metabolic products are still worth to be further investigated. 

Microemulsions have attracted considerable amount of interest as potential drug delivery vehicles, largely due to their simplicity of preparation, clarity and ability to be filtered and incorporate a wide range of drugs of varying solubility. Oil-in-water (o/w) microemulsion is the most suitable formulation, which is expected to increase the solubility by dissolving compounds with low water solubility into an oil phase. They can also enhance oral bioavailability by reducing the droplet size (< 100 nm), and hence increase the rate of absorption due to surfactant-induced permeability changes.

A variety of drugs that inhibit platelet function have been shown to decrease morbid events in patients with established cardiovascular atherosclerotic diseases, as evidenced by stroke or transient ischemic attacks, myocardial infarction, unstable angina or the need for vascular bypass or angioplasty. 

Clopidogrel selectively inhibits the binding of adenosine diphosphate (ADP) to its platelet receptor and the subsequent ADP-mediated activation of the glycoprotein GPIIb/IIIa complex, thereby inhibiting platelet aggregation. Clopidogrel also inhibits platelet aggregation induced by agonists other than ADP, by blocking the amplification of platelet activation due to the released ADP. However, clopidogrel does not inhibit phosphodiesterase activity. Clopidogrel acts by irreversibly modifying the platelet ADP receptor. Consequently, platelets exposed to clopidogrel are affected for the remainder of their lifespan. However, oral bioavailability of clopidogrel is very low (less then 50%), due to poor water solubility. Hence, the objective of this study was to enhance the solubility of clopidogrel by formulating it into a microemulsion.

## Experimental

*Materials*

Clopidogrel was received as a gift sample from Torrent Pharmaceutical Ltd. (Mehsana, India). Tween-80 and polyethylene glycol 400 were purchased from S.D. Fine Chemicals (Mumbai, India). Capmul MCM was kindly gifted by Abitec Corporation (USA). All the other chemicals and solvents were of analytical reagent grade and used without further purification.

*Selection of the oil phase*

Selection of the oil phase was based upon the maximum solubility of the drug. Different oils including Capmul MCM, Labrafac CC, cotton seed oil, sunflower oil and soybean oil were taken for solubility studies. 

*Selection of surfactant and co-surfactant*

The criteria for the selection of surfactant were its HLB value and non-toxic nature. Several surfactants including tween-80, Captex-355, Cremophor EL and Labrafil were screened. Co-surfactants were selected based on their capability to form stable microemulsion with relevant surfactants at a minimum concentration. Based on this, several co-surfactants including polyethylene glycol 400 (PEG 400), glycerol, polyethylene glycol 600 (PEG 600) and Transcutol P were screened.

*Pseudo-ternary phase diagram*

Pseudo-ternary phase diagrams were constructed to obtain the appropriate components, and their concentration ranges that resulted in a large existence area of microemulsion were chosen. In order to optimize the concentration of oil phase, surfactant and co-surfactant, different batches of varied concentration were prepared and titrated with distilled water till transparency persisted. Ternary phase diagram was prepared by using a constant ratio of surfactant to co-surfactant. Four ratios of surfactant (tween 80) and co-surfactant (PEG 400) were selected (1:1, 2:1, 3:1 and 4:1).

*Preparation of microemulsion*

Predetermined amounts of the drug were dissolved in the required quantity of oil. Surfactant and co-surfactant were added to the above mixture as a fixed ratio. Distilled water was added gradually with continuous stirring, which resulted in the formulation of a transparent and homogenous microemulsion. Parameters optimized for the preparation of microemulsion were the type and concentration of the oil phase, surfactant and co-surfactant.

*Characterization of microemulsion*

*(I) Transmittance test*

Stability of the microemulsion optimized formulation with respect to dilution was checked by measuring transmittance at 650 nm with a UV spectrophotometer (UV-1601-220X, Shimadzu).

*(II) Globule size and zeta potential measurements*

The globule size and zeta potential of the microemulsion was determined by dynamic light scattering, using a Zetasizer HSA 3000 (Malvern Instruments Ltd., Malvern, UK). 

*(III) Viscosity measurements*

Rheological behavior of the formulation was evaluated using a Brookfield LVDV ΙΙΙ+ cone and plate (CP) viscometer (Brookfield, USA), using the rheocal software, at a temperature of 30 ± 1°C.

*(IV) Electrical conductivity*

The water phase was added drop wise to a mixture of oil, surfactant and co-surfactant and the electrical conductivity of formulated samples was measured using a conductometer (CM 180 conductivity meter, Elico, India) at ambient temperature and at a constant frequency of 1 Hz.

*(V) Drug stability*

The optimized microemulsion was kept under cold condition (4-8 ^o^C), room temperature and at elevated temperature (50 ± 2 ^o^C). After every 2 months the microemulsion was analyzed for phase separation, % transmittance, globule size and % assay. 

*(VI) Drug solubility*

Drug was added in excess to the optimized microemulsion formulation as well as each individual ingredient of the formulation. After continuous stirring for 24 h at room temperature, samples were withdrawn and centrifuged at 6000 rpm for 10 min. The amount of soluble drug in the optimized formulation as well as each individual ingredient of the formulation was calculated by subtracting the drug present in the sediment from the total amount of drug added. The solubility of drug in microemulsion was compared with respect to its individual ingredients. 

*Drug release studies*

*(I) In-vitro drug release*

The diffusion studies were carried out, using a modified Franz diffusion cell. The receptor compartment was filled with 20 mL of pH 7.4 phosphate buffer. The donor compartment was fixed with cellophane membrane (cut off weight of 1000 Da), containing the microemulsion formulation (equivalent to 5 mg of clopidogrel) and the plain drug solution, separately. At predetermined time intervals samples were withdrawn from the receptor compartment and analyzed for drug content, using a UV spectrophotometer at 221.2 nm. 

*(II) Ex-vivo drug release*

*Ex-vivo *drug release into phosphate buffer pH 7.4 was studied using intestinal membrane within a Franz diffusion cell. Microemulsion formulation (equivalent to 5 mg) and the plain drug solution were placed in the donor compartment of two separate diffusion cells and the temperature of each cell was maintained at 37 ± 2 °C. The amount of drug released from the microemulsion formulation was estimated spectrophotometrically at 221.2 nm, by withdrawing samples from the receptor compartment at predetermined time intervals. 

## Results and Discussion

*Preparation of microemulsion*

The maximum amount of drug was found to dissolve in Capmul MCM (54.0 ± 1.27 mg/mL).Therefore, this oil was selected for microemulsion formulation. The required HLB value to form o/w microemulsion should be between 12-18 and the selection of surfactant was mainly based on this. Solubility of drug, as well as Capmul MCM was higher in tween 80. Co-surfactants were selected based on their capability to form a stable microemulsion with the relevant surfactants at a minimum concentration. Polyethylene glycol 400 (PEG 400) was selected for tween 80 containing microemulsions.

**Figure 1:**
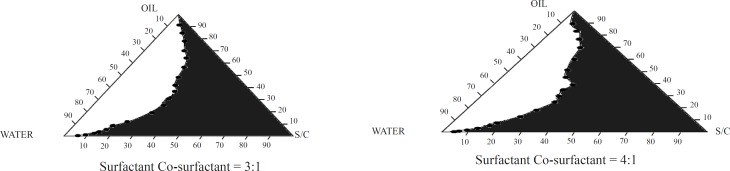
Ternary phase diagrams of microemulsions prepared

Pseudo-ternary phase diagrams were constructed to obtain the appropriate components and their concentration ranges that can result in a large microemulsion existence area. From the ternary phase diagrams shown in Figure 1, it was concluded that the highest microemulsion zone was achieved for the microemulsions containing tween-80/PEG 400 at a ratio of 4:1. A maximum amount of water uptake was found at 3% Capmul MCM and 35% tween 80/PEG 400 (4:1) concentration.

*Characterization of microemulsions*

*(I) Transmittance test*

The percentage of transmittance of the optimized microemulsion formulation, as well as its 100 times dilution with 0.1N HCl, was checked at 650 nm and found to be 99.76 ± 0.038 and 99.82 ± 0.051, respectively. 

*(II) Globule size measurement*

**Figure 2:**
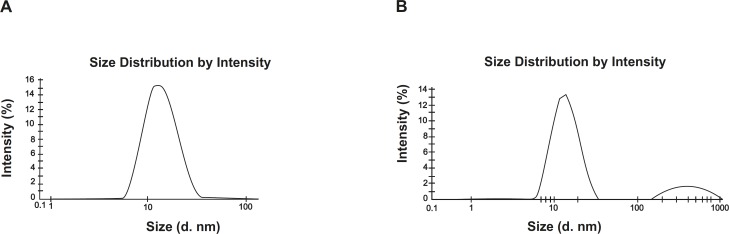
Average globule size of the optimized microemulsion (A) and its diluted form (100 times with 0.1N HCl) (B).

The optimized Capmul microemulsion showed very small particle size (*i.e*. 12.3 nm) and upon 100 folds dilution with 0.1N HCl and storage for 3 h, it showed very little change in particle size (*i.e*. 15.8 nm). The value of polydispersity index (PI) of both samples were found to be below 1.0, suggesting that upon dilution with gastric fluid in body, the optimized microemulsion formulation remains stable and will not convert into macroemulsion. Results of globule size have been shown in Figure 2. 

*(III) Zeta potential measurements*

**Figure 3:**
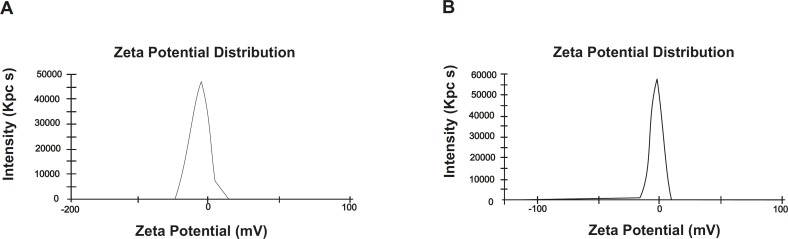
Zeta potential of the optimized microemulsion (A) and its 100 times diluted form (B), respectively

Zeta potential results of the optimized microemulsion and its diluted form (100 times diluted with 0.1N HCl) have been shown in Figure 3, and were found to be -6.34 mV and -3.02 mV, respectively. Aggregation is not expected to take place, due to the slightly negative charge of the droplets. 

*(IV) Viscosity measurements*

All microemulsion samples were found to have rather low viscosities, ranging from 40 to 90 cps. There was an increase in viscosity from 79.46 to 86.25 cps, with an increase in the water content from 0 to 10%. However, after the addition of 10% aqueous phase, the viscosity of the system gradually decreased. It was also observed that the viscosity of the system gradually and slowly decreased with an increase in the aqueous phase when 10% < Фw < 70%, followed by a decrease beyond 70%. This may be due to the fact that the system transforms from w/o (when Фw >10%) through bicontinuous structure (10% < Фw < 70%) to an o/w system. 

*(V) Electro-conductivity measurement*

The electrical conductivity (σ) was almost zero, as long as the percentage of aqueous phase was less than 8. During the aqueous phase titration, up to 33% water content, electrical conductivity increased rapidly. When the percentage content of the aqueous phase was above 33%, the conductivity of the system was not affected significantly with further addition of the aqueous phase. Hence, the middle phase microemulsion was kept between 8% to 33% aqueous phase addition. Above 33% aqueous phase addition, it is converted to an o/w microemulsion. Since the optimized Capmul microemulsion formulation contained 75% of the aqueous phase, hence, the structure of the optimized Capmul microemulsion is an o/w structure.

*(VI) Drug solubility*

**Figure 4:**
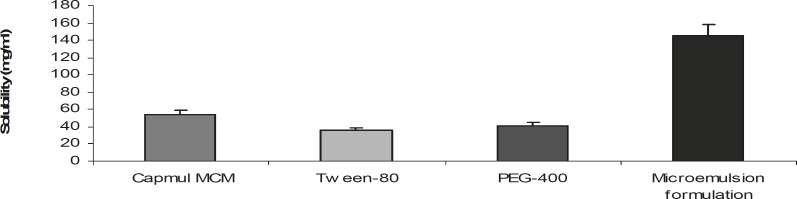
Solubility of clopidogrel in different components of microemulsion and the optimized microemulsion (n = 3; mean ± SD).

Solubility of the drug in microemulsion formulation and the individual ingredients of the microemulsion have been shown in Figure 4. The results indicate an enhanced solubility of clopidogrel in the optimized formulation, when microemulsion was compared to its respective individual ingredients.

*(VII) Stability studies*

Results of temperature stability studies on the optimized microemulsion has been recorded in Table 1. The optimized microemulsion was also subjected to centrifugal stability studies. Results obtained from both the stability studies indicated that the optimized Capmul MCM based microemulsion was stable up to 6 months. 

**Table 1:** Effect of temperature on stability of the optimized microemulsion formulation (n = 3; mean ± SD).

**Temperature ( ** ^°^ **C) **	**Phase separation **		**% transmittance **		**Particle size (nm) **		**% of Assay **	
	**After 4 months **	**After 6 ** **months **	**After 4 ** **months **	**After 6 ** **months **	**After 4 months **	**After 6 months **	**After 4 months **	**After 6 ** **months **
**2 -8 **°**C **	No	No	98.2 ± 0.8	97.8 ± 2.4	17.9 ± 2.4	18.3 ± 1.8	98.3 ± 1.5	97.8 ± 2.9
**Room temperature ** **(25±2 **°**C) **	No	No	99.1 ± 1.2	98.8 ± 1.3	17.2 ± 2.1	19.1 ± 3.1	99.4 ± 1.9	99.1 ± 2.1
**Elevated temperature (50 ± 2 ** ^°^ **C) **	No	No	99.1 ± 0.8	98.2 ± 1.5	18.2 ± 2.6	19.7 ± 3.2	99.2 ± 1.8	98.6 ± 1.7

*(VIII) In-vitro drug release studies*

**Figure 5:**
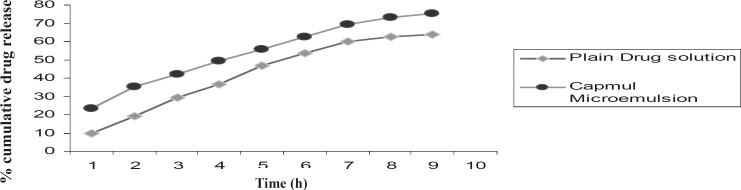
*In-vitro *drug release profile of clopidogrel from the microemulsion formulation and the plain drug solution (n = 3).

Release profile of clopidogrel was carried out from the optimized microemulsion formulation, as well as the plain drug solution, and the results have been shown in Figure 5. After 9 h, drug released from the plain solution and the Capmul microemulsion was 63.76% and 75.53%, respectively. From this study, it can be concluded that the extent of diffusion of clopidogrel from the microemulsion formulation is greater than the plain drug solution. The Capmul microemulsion showed a faster clopidogrel release, than the plain solution, due to its smaller microemulsion droplet size (12.3 nm).The sustained drug release observed may be due to the practically insoluble nature of clopidogrel in water.

*(IX) Ex-vivo release studies*

**Figure 6:**
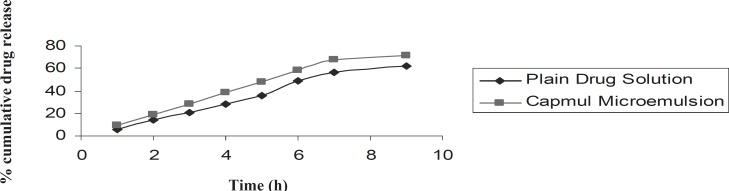
*Ex-vivo *release profile of clopidogrel from the microemulsion formulation and the plain drug-containing solution (n = 3).

*Ex-vivo *release profile of clopidogrel was carried out on the optimized microemulsion formulation, as well as the plain drug solution and the results have been shown in Figure 6. After 9 h, the amount of drug released from the plain solution and the microemulsion formulation was 61.90% and 71.20%, respectively. From this study, it can be concluded that the extent of diffusion of clopidogrel from the Capmul microemulsion is greater than the plain solution, which may be due to the penetration enhancing effect of surfactant and co-surfactant present within the microemulsion formulation, aiding passage of the drug molecule through the intestinal membrane. The rather small globule size of the microemulsion formulation can also help this matter. Overall, the microemulsion formulation showed a higher extent of absorption than the plain drug solution. The sustained profile of drug release observed may be due to the fact that drug is present within the oil phase and hence has a higher partition coefficient. 

## Conclusion

This study demonstrates that the microemulsion formulation can be employed to enhance the solubility of poorly water soluble drugs like clopidogrel. The optimized microemulsion formulation containing Capmul MCM (3%), tween 80 (28%), PEG 400 (7%) and distilled water is a transparent and low viscosity system, with a particle size of 27.9 nm. The stability studies confirmed that the optimized microemulsion was stable for six months. Results from the in-vivo studies revealed that the developed microemulsion formulation possessed a higher rate and extent of absorption, compared to the plain drug solution. The solubility profile of drug indicated that the microemulsion formulation can enhance solubility by 80.66 folds, compared to distilled water (pH = 7.4), which may increase the oral bioavailability of clopidogrel. However, further studies on animals and human being are needed to be performed before this formulation can be commercially exploited. 
